# Will the Economic Recession Inhibit the Out-of-Pocket Payment Willingness for Health Care?

**DOI:** 10.3390/ijerph17030713

**Published:** 2020-01-22

**Authors:** Yuhang Zheng, Zhehao Huang, Tianpei Jiang

**Affiliations:** 1 School of Finance and Collaborative Innovation Center of Scientific Finance & Industry, Guangdong University of Finance & Economics, Guangzhou 510320, China; yhzheng@gdufe.edu.cn; 2 Guangzhou International Institute of Finance, Guangzhou University, Guangzhou 510405, China; 3 School of Information Science and Technology, ShanghaiTech University, Shanghai 201210, China; jiangtp@shanghaitech.edu.cn

**Keywords:** economic recession, health care, out-of-pocket payment willingness, financial crisis

## Abstract

We used an individual regression and panel data regression method to analyze the samples of 60 countries from 2000 to 2016 to study the impact of the economic recession on residents’ out-of-pocket payment willingness for health care. Although we found an increase in the willingness during the economic recession in most countries, we couldn’t find significant evidence of a positive relationship between the economic recession and such willingness. We discovered that the relationship differentiates in different countries, which mainly depends on the differences in the medical systems and degree of economic development. By controlling individual differences in countries, we found that the economic recession inhibited the out-of-pocket payment willingness for health care. Especially after the impact of the financial crisis in 2008, the cumulative effect of the economic recession and the aftershock of financial crisis was discovered, which significantly inhibited residents’ willingness. In addition, we verified that the economic recession inhibited the out-of-pocket payment willingness by reducing employee compensation in specific types of countries.

## 1. Introduction

Health is a human right and is a prerequisite for the economic and social development of any country. However, as the service and products provided by the healthcare industry are often affected by the market, their prices and quality also change. Therefore, fluctuations in economic and financial markets have impact on the providers and users of health care services and products, which will ultimately influence people’s health.

If a country wants to ensure a healthy population, it must run a healthy health care system and make sure that every resident is able to pay for these services. During economic recession, many health care systems around the world provide funding primarily through out-of-pocket payments, which enables governments to minimize their healthcare costs [1,2,3]. Out-of-pocket payment is an un-reimbursable way, which prevents a large number of residents from obtaining health services. Since these people cannot afford health care expenditures for themselves, they often raise debt to meet the necessary medical health needs. This fact leads to catastrophic health expenditures and for people to impoverish themselves [3,4,5].

Due to the cyclical economic recession, more and more literature started to focus on the impact of health care expenditures on the national economy and household finances, and the research objects included groups of countries [6,7] and individual countries [8,9]. All the findings were examined at the microeconomic level by considering the impact of health care expenditures on the individuals, households, or on the macroeconomic level. The main conclusion is that catastrophic health expenditures may force households to reduce consumption of other minimum needs, which may cause productive asset sale or high levels of debt. Then, it leads to impoverishment and ultimately affects health levels [10,11,12,13].

This paper empirically analyzes the impact of the economic recession on the out-of-pocket payment willingness for health care. The main contributions are as follows. (i) Instead of focusing on certain regions or a single economy, our study covers 60 countries from 2000 to 2006. The dataset is divided into different groups of categories where the differences are analyzed. (ii) Instead of focusing on a certain type of global economic crisis, we use the Hodrick-Prescott (HP) filtering method to extract the economic cycle of each country and label the period of economic recession. (iii) We also pay attention to the impact during the post-crisis and the economic recession, which expands the research horizon of the changes in residents’ out-of-pocket payment willingness for health care. (iv) By the mediating effect model, we test that the economic recession inhibits residents’ out-of-pocket payment willingness for health care by reducing the level of salary and income.

The rest of the paper is organized as follows. Section 2 is the literature review. Section 3 describes the methods of empirical analysis. Section 4 shows data and the main results of empirical analysis. The conclusion is in Section 5.

## 2. Literature Review

Some research focused on the relationship between economic growth and health care expenditure. The main research objects included income elasticity of health care, policy implementation of medical financing, distribution characteristics of medical resources, and the leading relationship between medical expenditure and economic growth [14,15,16,17,18]. The relationship between economic growth and health expenditure is based on two basic assumptions. First, health care is a luxury with the attributes of commodities, whose price should be determined by the market. Second, health care is also a necessity, implying that the health care sector should be intervened by government policies to meet the needs of different groups.

To protect the citizens who need medical services, countries have established health insurance systems to address the issue of health risk allocation. All medical systems require anticipated payments, which influences the decisions of citizens on the health-care payment, although medical systems are designed differently around the world [19,20]. As a supplement to the universal health insurance system, many countries rely heavily on patients to have out-of-pocket payment for health care. Excessively high out-of-pocket medical payment can lead to catastrophic expenditure [4,21,22]. Even for countries with tax-based universal health insurance systems, we do not have sufficient evidence to show the impact of economic recession on health care payments [23,24,25,26,27].

Some empirical studies prove that the economic recession had a significant impact on health care expenditure. By studying the changes in health care expenditures in Greece and Serbia during the financial crisis, researchers found that the health markets of these two countries had different performances with fiscal austerity caused by the global economic recession [28]. Greece was forced to reduce total medical and pharmaceutical expenditures, resulting in severe out of pocket payments. As an emerging market, Serbia successfully maintained a 19% increase in health expenditure and even a 25% increase in medical expenditure, despite of the severe impact of the global economic recession. In [29], it showed that the global financial crisis in 2008 had a full impact on Italian families in the field of health care. Residents gradually reduced private health expenditures and asked the public sector to share health costs. Empirical analysis given by [30] showed the relationships among life expectancy and public and private health expenditures, where there is a positive correlation between public health expenditure and life expectancy and there is an important impact of private expenditure on life expectancy. The work [31] investigated the impact of the Greek economic recession on the health of newborns and finds that birth weight and time of pregnancy are usually procyclical, while the risks of low birth weight and of preterm birth are countercyclical. The study also reports the heterogeneity of the relationship between economic cycle fluctuations during pregnancy and newborn health in different socioeconomic groups.

Although the previous literature provided some hints on the relationship between the economic recession and the payment of health care costs, there is no country-specific study on this relationship. Therefore, by grouping the sample countries according to the differences in the medical system and the degree of economic development, we studied the significant impact of economic recession on the out-of-pocket payment willingness for health care.

## 3. Methodology

We referred to the local projection model in [32] and introduced macroeconomic control variables and established the model to illustrate the impact of economic recession on the out-of-pocket payment willingness for health care. Here, some symbols in the statistical model need to be explained. 

For *N* number of countries or regions, $y_{i.t+h}$ represents the level of out-of-pocket payment for health care in *h* steps ahead at time *t* for country *i*. Here, denote by $PW_{i,t}$, the change of out-of-pocket payment for health care over time, that is,

(1) $$PW_{i,t}=a_{i}+\beta_{i}C_{i,t}+\sum_{k=1}^{K}\delta_{i,t}x_{i,t}^{k}+\varepsilon_{i.t+h}$$

In order to achieve our research goals, we focused on the symbolic direction and significance of the parameter $\beta_{i}$, which represents the impact of the economic recession on the out-of-pocket payment willingness for health care by eliminating the control variables. A positive parameter $\beta_{i}$ implied that economic recession boosts out-of-pocket payment willingness for health care and a negative parameter $\beta_{i}$ implies the other way. As is suggested in [32], the benchmark model used to calculate local predictions can be fitted by simple regression by standard regression packages. In addition, we also considered the heterogeneity among countries. Thus, we divided the countries into different groups to analyze, and describe the distribution characteristics of the effect of the economic recession between groups.

Then, we analyzed empirically the impact of the economic recession on out-of-pocket payment willingness for health care with the shock of the financial crisis. There was a strong correlation or synchronization between economic cycles and financial fluctuations [33,34,35,36] and residents’ medical expenditure may be affected by the relationship. Especially after the financial crisis in 2008, the cyclic economic recession and the aftershock of the financial crisis showed the cumulative effect on the out-of-pocket payment willingness for health care. 

Hypothesis 2: The aftershock of the financial crisis strengthened the inhibitive effect of economic recession on the out-of-pocket payment willingness for health care.

Thus, assume that $FC_{t}$ is the indicator to label before and after of financial crisis in 2008. The value is 1, if the time period is after financial crisis and 0 otherwise. Take $Country_{n}$ as a dummy variable to control heterogeneity of the individual country. Then, we introduce $BC_{i,t}\times FC_{t}$ into the model and the panel data model is constructed as follows.

(2) $$PW_{i,t}=a+\beta BC_{i,t}+\gamma BC_{i,t}\times FC_{t}+\sum_{k=1}^{K}\delta_{k}x_{i,t}^{k}+\sum_{n=1}^{N}Country_{n}+\varepsilon_{i,t}$$

Here, parameter β represents the degree of common impact of the economic recession on the out-of-pocket payment willingness for health care in countries and γ explains the impact of both economic recession and financial crisis on the willingness. Parameter γ is negative, if financial crisis strengthens the inhibition of economic recession on the out-of-pocket payment willingness for health care and is positive if the economic recession promotes the willingness after the financial crisis.

Hypothesis 3: Economic recession inhibited the out-of-pocket payment willingness for health care by reducing residents’ income.

The out-of-pocket payment willingness for health care had a positive relationship with the actual income of residents [37] and the income of residents (wage level) and economic fluctuations are procyclical [38]. In order to analyze the impact of the economic recession on the out-of-pocket payment willingness for health care, based on model (1), we considered the changes in employee wage. That is, we introduced the interaction term of the employee wage index and the economic recession to conduct regression analysis to test how the economic recession affects the out-of-pocket payment willingness for health care by affecting the income level of residents. Here, we needed to test whether the economic recession had a significant impact on employee wage and the model, and is as follows,

(3) $$wage_{i,t}=a+\beta BC_{i,t}+\sum_{k=1}^{K}\delta_{i}x_{i,t}^{k}+\sum_{n=1}^{N}Country_{n}+\varepsilon_{i,t}$$

Then, we set up a panel data model containing the interaction terms of economic recession and employee wage as follows,

(4) $$PW_{i,t}=a+\beta BC_{i,t}+\gamma BC_{i,t}\times wage_{i,t}+\sum_{k=1}^{K}\delta_{i}x_{i,t}^{k}+\sum_{n=1}^{N}Country_{n}+\varepsilon_{i.t}$$

Assume that model (1) shows how economic recession reduces employee wage. Positive parameter γ in model (2) implies that economic recession inhibits the out-of-pocket payment willingness for health care by reducing actual income and negative parameter γ implies that economic recession increases the spending on health care, even though it reduces the actual income.

## 4. Empirical Analysis

### 4.1. Data Description

**Figure A1:**
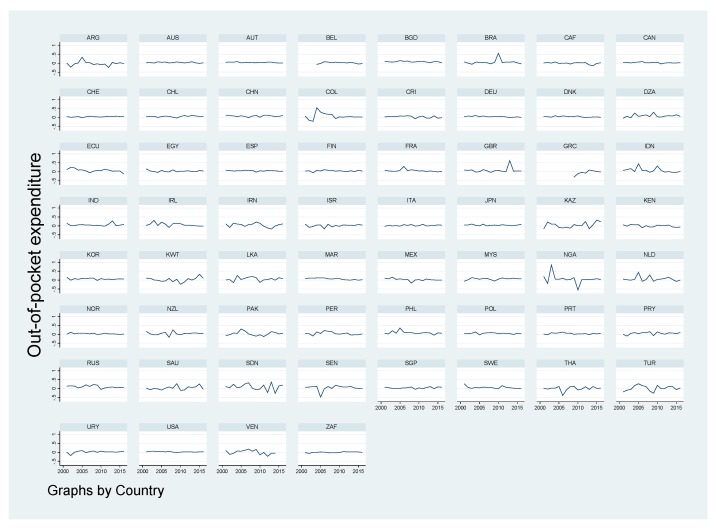
Out-of-pocket expenditure change.

The out-of-pocket payment willingness of residents for health care can be measured by the amount of per capita spending after eliminating inflation. If the out-of-pocket payment for health care increases year-on-year, we say the residents’ willingness increases. Otherwise, we say the willingness decreases. The time series of the annual per capita out-of-pocket payment indicators for each country was obtained from the Health Nutrition and Population Statistics of the World Bank database. Data from 2000 to 2016 for each country allowed us to conduct cross-country comparative study to the greatest extent. In the empirical process, we used the logarithmic value of the original data. The trend of willingness in various countries is shown in Figure A1.

The selection of control variables was mainly based on research [39,40], and the availability of control variable data was also considered. The selected variables included: (1) the logarithm of GDP per capita, in order to control the development level of the country; (2) the average saving, in order to control the impact of personal savings; (3) the unemployment rate, in order to control the impact of unemployment on consumer spending; (4) the loan interest rate, in order to control the development of the financial credit market, (5) the M2/GDP indicator, in order to control the expansion of the money market, and (6) the CPI indicator, in order to control domestic consumer inflation. These control variables were obtained from the World Bank’s World Development Indicators (WDI) database.

In the empirical analysis sample, we selected 60 countries which produced the major proportion of the world’s GDP. Also, relevant data on personal health care payment was available for these countries. For each country, the number of periods of economic recession was no less than 2 and the number of control variables was no less than 4.

**Figure A2:**
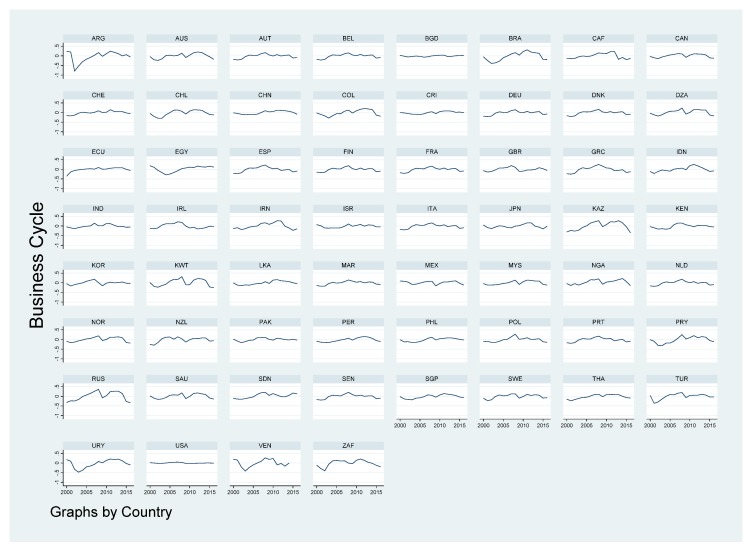
Economic cycle.

Referring to [41], we identified the economic cycles of countries based on non-trending time series of GDP. Thus, we used the band-pass filter proposed by [42] to extract the periodic components of the time series of GDP. This method minimized the degree of deviation from the trend and limited the smoothness of the trend at the same time. Finally, the method separated the periodic components of the time series. To avoid the statistical error brought by this method [43], we had the following setting. The value is 0, if the economic cycle (BC) is identified as the expansion of the economic cycle and 1, otherwise. By data of economic cycle (see Figure A2), we can construct the economic recession dummy variable $BC_{i,t}$ for each country *i* at time *t*.

### 4.2. Impact of the Economic Recession

Using the least squares method to estimate model (1), we could obtain the corresponding coefficients corresponding to the out-of-pocket payment willingness for health care during the economic recession.

Table 1 reports the impact of the economic recession on the out-of-pocket payment willingness for health care. The table shows that the economic recession decreased the out-of-pocket payment willingness for health care in 33.3% of countries. For the other countries, residents increased their out-of-pocket payment willingness for health care, which may be related to residents’ awareness of health care. However, the economic recession had a significantly negative impact on 8.3% of countries and significantly positive impact on 16.7% of countries, respectively, on the out-of-pocket payment willingness for health care.

**Figure 1:**
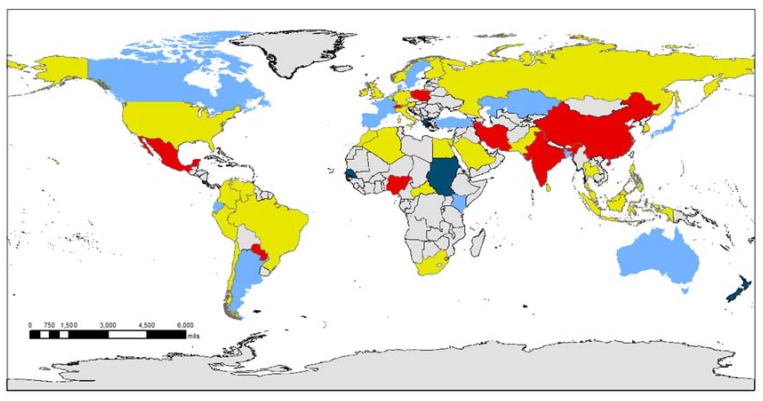
The effect of the economic recession. Note: The red (yellow) countries are those that showed a significant (not significant) increase in the willingness as a result of the recession cycle, while the dark blue (light blue) countries are those that showed a significant (not significant) decrease in the willingness due to these crises.

Figure 1 shows how the impact of the economic recession on the out-of-pocket payment willingness for health care varies across geographic regions. The red (yellow) countries are those that show a significant (non-significant) increase in the willingness as a result of the recession cycle, while the dark blue (light blue) countries are those that showed a significant (non-significant) decrease in the willingness due to these crises. According to Panel A in Table 1, countries, in which the economic recession significantly inhibited the out-of-pocket payment willingness for health care, include Costa Rica, Greece, New Zealand, Sultan, and Senegal.

Now we further analyze the impact of the economic recession on the out-of-pocket payment willingness for health care from the perspectives of the medical insurance system and geography. The specific classifications of each country are shown in Appendix A. There are types of medical insurance systems, Compulsory Health Insurance System (CHIS) and non-Compulsory Health Insurance System (non-CHIS). We should mention that Compulsory Health Insurance System is operated by Nation Health Service (NHS) and Compulsory Social Health Insurance (CSHI). Countries with Non-Compulsory Health Insurance System usually implement commercial insurance systems, savings medical insurance systems, or hybrid medical insurance systems. Panel B in Table 1 shows that after the economic recession, the out-of-pocket payment willingness for health care decreases in 37% of CHIS countries and the willingness decreases in 31.3% of non-CHIS countries. However, this effect is statistically significant in only 7.4% of CHIS countries and 9.1% of non-CHIS countries respectively.

To conduct the geographic analysis of the impact of the economic recession on the out-of-pocket payment willingness for health care, we classified countries according to the 2017 National Income Classification published by the World Bank. We treated high-income countries as developed market countries and the other countries as emerging market countries. Panel D in Table 1 report that the economic recession inhibited willingness among 41.4% of high-income countries (of which 6.9% are facing significant inhibition). For low- and middle-income countries, the proportion of the impact was 25.8% (9.7% of which is significant). 

According to the analysis [44], we believe that economic development was not the only reason to explain the differences in the out-of-pocket payment willingness among countries. We were also aware that social-behavioral factors would affect residents’ habits of participation in insurance, and the prevalence rate of un-insurance also varies in different regions [45]. Therefore, geographic or cultural differences may explain the different reactions of residents to the cyclic economic recession in the out-of-pocket payment willingness in different countries. To analyze this potential difference in the willingness, we divided the countries into different regional clusters, that is, Africa, Asia, Europe, Latin America, and Anglo-Saxon (Since UK has already appeared in the set of European, it will be included in the set of Anglo-Saxon countries). By panel D in Table 1, in countries except Anglo-Saxon, the willingness did not get excessively inhibited after the recession of the economic cycle. Especially in Asian countries, there was no significant inhibition of individual’s willingness. On the contrary, the out-of-pocket payment willingness for health care was promoted in 26.3% of countries after the recession of the economic cycle.

### 4.3. Impact of the Financial Crisis

Next, we further studied whether the economic recession inhibited the overall out-of-pocket payment willingness for health care. By analyzing the estimation results of the panel data model, the effect of the economic recession could be obtained. At the same time, the dummy variable of the financial crisis (FC) was introduced into the model and the cumulative effect of the financial crisis and the economic recession could be observed. The result is shown in Table 2.

Panel A in Table 2 report the impact of the economic recession in the economic cycle on the out-of-pocket payment willingness for health care using the full sample. It showed that the coefficient of the impact of economic recession economic cycle (BC) is −0.0132 with significant level 10%, implying that economic recession inhibits the willingness. After introducing the indicator of the impact of financial crisis FC, the coefficient of BC turned to 0.0138 non-significantly and the coefficient of cumulative effect of economic recession and financial crisis turned to −0.0375 with significant level 1%. This fact implies that the impact of economic recession on willingness became more significant after the financial crisis. In addition, the cumulative effect of the economic recession and financial crisis greatly reduced the payment ability of residents, especially the payment ability for health care.

According to the model involved in the health insurance system, we can see that after the financial crisis, the economic recession had a different impact on out-of-pocket payment willingness for health care in different countries. Panel B in Table 2 report the estimated results for both CHIS and non-CHIS countries. In CHIS countries, the economic recession did not inhibit the out-of-pocket payment willingness for health care, since the compulsory health insurance system with taxation or compulsory payment of medical insurance reduces the impact on the willingness of the economic recession. Under such a medical system, health care expenditure meets the basic medical needs, and thus the out-of-pocket payment was changed significantly. For Non-CHIS countries, the health insurance system gets along with the trend of the economic cycle. Thus, economic recession inhibited the out-of-pocket payment willingness and the aftershock of financial crisis even strengthened the inhibitive effect.

We now discuss the difference in the out-of-pocket payment willingness for health care with respect to geographical difference in the economic recession after the financial crisis. Panel C in Table 2 showed the estimated results in both OECD and non-OECD countries. For the OECD countries, economic recession significantly inhibited the out-of-pocket willingness for health care with coefficient −0.0306 and this effect did not change after the financial crisis. The financial crisis even worsened the inhibitive effect of the economic recession. The economic recession in non-OECD countries did not inhibit the willingness, but its promotion effect was not significant. After the shock of the financial crisis, the economic recession significantly increased the out-of-pocket payment willingness for health care. With the opposite effect, the overall effect of increasing willingness may not be achieved.

Panel D in Table 2 report the impact of the economic recession and the financial crisis on the out-of-pocket payment willingness to pay for health care among high-income countries and low- and middle-income countries. The economic recession in high-income countries increased the out-of-pocket payment willingness for health care, and the willingness got stronger after the financial crisis from 0.0027 to 0.0462. The cumulative effect of economic recession and financial crisis inhibited such willingness. In low- and middle-income countries, economic recession alone decreased the willingness. However, after the financial crisis, the willingness significantly increased. Similar to the situation of high-income countries, the cumulative effect of economic recession and financial crisis inhibited such willingness.

Panel E in Table 2 report that the economic recession and financial crisis in different regions with political and cultural heterogeneity had an impact on the out-of-pocket payment willingness for health care. Except for Asian countries, economic recession inhibited the willingness, especially in European countries. After financial crisis, except for European countries, the economic recession increased the willingness non-significantly. With the cumulative effect of economic recession and financial crisis, the out-of-pocket payment willingness for health care was inhibited in the other regions except for European countries.

### 4.4. Test of the Influential Channel

Using models (2) and (3), we studied the influential mechanism of economic recession on inhibiting the out-of-pocket payment willingness for health care in the wage distribution. Table 3 showed the test results of the impact channels (Panel A), and the test results of heterogeneity in different groups of countries (Panel B, Panel C, Panel D, Panel E).

Panel A in Table 3 report the impact of the economic recession on the out-of-pocket payment willingness for health care by affecting employee compensation on the full sample. The table verifies the pro-cyclical relationship between employee compensation and the economic cycle. That is, employee wage decreased in the economic recession. The corresponding coefficient of the interaction term of economic recession and employee compensation in the regression is 0.0019 with significant level 5%. It implies that with the increase in employee compensation, disposable income increases, and then the out-of-pocket payment ability and willingness for health care also increased. During the economic recession, residents’ ability to pay for health care decreased with the reduction of employees’ compensation, which inhibited their out-of-pocket payment willingness. This fact verifies that reduced employee compensation is an influential way for the recession to inhibit the out-of-pocket payment willingness for health care.

Panel B in Table 3 report whether the economic recession affected the out-of-pocket payment willingness for health care by affecting employee compensation under countries with different health insurance systems. For CHIS countries, the effect was non-significant. Most of CHIS countries are small and developed with comprehensive social security systems and compulsory health insurance systems, few of which have periodic unemployment problems. For non-CHIS countries, this effect was significant. In non-CHIS countries with a non-compulsory participation insurance system, the out-of-pocket payment willingness for health care reduced as wages reduced.

Panel C and panel D in Table 3 report the impact of the economic recession on the out-of-pocket payment willingness for health care by affecting employee compensation by grouping of different geographical conditions and by grouping of high-income countries and low- and middle-income countries. In Panel B, for non-OECD countries, by reducing employee compensation, the economic recession inhibited the out-of-pocket payment willingness for health care significantly. However, for OECD countries this impact was not significant. Although the interaction coefficient is significant, it cannot explain that the economic recession reduced such willingness by reducing wage compensation. Panel C concludes that the reduction in wage in high-income countries cannot explain whether the economic recession inhibited the out-of-pocket payment willingness for health care, but the effect was significant in low- and middle-income countries.

Penal E shows that for countries in different regions with political and cultural heterogeneity, the impact of the economic recession on the out-of-pocket payment willingness for health care was different. For countries in Africa, reduction in employees’ compensation decreased such willingness in the economic recession. For Asian countries, although the economic recession significantly reduced employees’ compensation, it did not significantly affect the willingness. For the countries of Europe and Anglo-Saxon, although the impact of employee compensation on the willingness was significant in a recession, employee compensation did not decline in the economic recession. For Latin America, the economic recession increased employee compensation and further increased willingness non-significantly. Thus, this conclusion cannot overturn the basic conclusion that the economic recession inhibited the out-of-pocket payment willingness for health care by reducing employee compensation.

## 5. Conclusions

Will the economic recession inhibit the out-of-pocket payment willingness for health care? We answered this question in a linear regression framework. Using a local regression model, we analyzed the impact of the economic recession on personal health care expenditure in 60 countries from 2000 to 2006. At the same time, we used the panel data model to conduct an empirical analysis to study the impact of the economic recession in full sample. We further analyzed the impact of the economic recession on the out-of-pocket payment for health care after financial crisis in 2008.

Using annual data from 60 countries from 2000 to 2016, we obtained some empirical facts. In the full sample, no significant evidence showed that the economic recession inhibited or promoted the out-of-pocket payment willingness for health care. We could see that the economic recession inhibited the out-of-pocket payment willingness for health care, although the effect on individual countries was non-significant. This fact led us to explore how the completeness of the health care market or the leading role of the public sector in each country affected the out-of-pocket payment willingness.

Then, we further eliminated individual heterogeneity. Under different grouping conditions, we found that the inhibition effect of the economic recession was different. For non-CHIS countries, European countries, and Anglo-Saxon countries, the economic recession inhibited the willingness significantly.

In addition, we found that the cumulative effect of the economic recession and financial crisis significantly inhibited the out-of-pocket payment willingness for health care. The conclusion is robust under all grouping conditions. The financial crisis exacerbated the impact of the economic recession on the inhibition of personal health care expenditures [24]. During economic recession, residents reduced personal expenditures on health care, which ultimately affected people’s health.

Finally, we tested how the economic recession inhibited the out-of-pocket payment willingness for health care by affecting employees’ compensation. That is, the economic recession reduced the willingness by reducing the wages of residents. For CHIS, OECD, high income levels, and European or Anglo-Saxon countries, reducing employee compensation may not have led to the fact of the inhibition of willingness during the economic recession, since a more developed social security system guaranteed the basic medical needs in these countries. Conversely, in non-CHIS, non-OECD, low- and middle-income, or in African countries, economic recession significantly inhibited the out-of-pocket payment willingness for health care by reducing wages.

## Figures and Tables

**Table 1:** The effect of the cyclic economic recession.

**Panel A. Total Sample**										
	N					P				
NS	25%					50%				
S	8.3%					16.7%				
**Panel B. CHIS vs. Non-CHIS**										
	CHIS					Non-CHIS				
	N			P		N		P		
NS	29.6%			55.6%		22.2%		45.5%		
S	7.4%			7.4%		9.1%		24.2%		
**Panel C. OECD vs. Non-OECD**										
	OECD					Non-OECD				
	N					P				
NS	33.3%			48.1%		18.2%		51.5%		
S	7.4%			11.1%		9.1%		21.2%		
**Panel D. World Bank High Income vs. Middle/Low Income Level**										
	High income					Middle/Low income				
	N					P				
NS	34.5%			44.8%		16.1%		54.8%		
S	6.9%			13.8%		9.7%		19.4%		
**Panel E. Regional Clustering**										
	Africa		Asia		Europe		Latin America		Anglo-Saxon (excluding UK)	
	N	P	N	P	N	P	N	P	N	P
NS	12.5%	50%	15.8%	57.9%	33.3%	50%	27.3%	45.6%	50%	25%
S	25%	12.5%	0%	26.3%	5.6%	11.1%	9.1%	18.2%	25%	0%

Note: The economic recession has the negative or positive impact on the out-of-pocket payment willingness for health care. For each group, symbol N, P, NS, and S stand for negative, positive, non-significant, and significant, respectively. Panel A is the estimated result of the full sample. Panel B divides the full sample into two sub-samples, that is, Compulsory Health Insurance System (CHIS) and Non-CHIS countries. Panel C divides the full sample into two sub-samples, that is, OECD and Non-OECD countries. Panel D divides the full sample into high-income and low-middle-income countries according to the World Bank’s classification criteria. Panel E is divided by regions.

**Table 2:** The effect of the cyclic economic recession under a financial crisis impact.

**Panel A. Total Sample**										
BC	−0.0132 * (0.0075)					0.0138 (0.0124)				
BC × FC						−0.0375 *** (0.0137)				
**Panel B. CHIS vs. Non-CHIS**										
	CHIS					Non-CHIS				
BC	0.0048 (0.0114)			0.0231 (0.0180)		−0.0264 *** (0.0100)		0.0075 (0.0169)		
BC × FC				−0.0265 (0.0201)				−0.0466 ** (0.0188)		
**Panel C. OECD vs. Non-OECD**										
	OECD					Non-OECD				
BC	−0.0306 *** (0.0087)			−0.0273 * (0.0144)		0.0027 (0.0118)		0.0462 ** (0.0195)		
BC × FC				−0.0044 (0.0157)				−0.0608 *** (0.0218)		
**Panel D. World Bank High Income vs. Middle/Low Income Level**										
	High income					Middle/Low income				
BC	0.0027 (0.0118)			0.0462 ** (0.0195)		−0.0036 (0.0123)		0.0519 ** (0.0204)		
BC × FC				−0.0608 *** (0.0218)				−0.0780 *** (0.0230)		
**Panel E. Regional Clustering**										
	Africa		Asia		Europe		Latin America		Anglo-Saxon (excluding UK)	
BC	−0.0187 (0.0289)	0.0048 (0.0465)	0.0147 (0.0135)	0.0375 * (0.0217)	−0.0356 *** (0.0117)	−0.0514 *** (0.0189)	−0.0281 (0.0182)	0.0499 (0.0305)	−0.0314 * (0.0169)	0.0003 (0.0240)
BC × FC		−0.0341 (0.0526)		−0.0318 (0.0236)		0.0222 (0.0208)		−0.1113 *** (0.0354)		−0.0485 * (0.0266)
Control variables	YES									
Virtual variable	YES									

Note: Panel A is the estimated result of the full sample. Panel B divides the full sample into two sub-samples, that is, Compulsory Health Insurance System (CHIS) and Non-CHIS countries. Panel C divides the full sample into two sub-samples, that is, OECD and Non-OECD countries. Panel D divides the full sample into high-income and low-middle-income countries according to the World Bank’s classification criteria. Panel E is divided by regions. BC and FC represent economic recession in the economic cycle and financial crisis, respectively. All models incorporate control variables and treat countries as dummy variables. Each subsample gives two columns of empirical results. The first column of explanatory variables includes economic recession variables and control variables, and the latter column introduces interaction variables of economic recession and financial crisis. Standard errors in parentheses *** *p* < 0.01, ** *p* < 0.05, * *p* < 0.1.

**Table 3:** The effect of the cyclic economic recession by wage.

**Panel A. Total Sample**										
	wage					PW				
BC	−0.0706 *** (0.0255)					−0.0633 *** (0.0208)				
BC × wage						0.0019 ** (0.0008)				
**Panel B. CHIS vs. Non-CHIS**										
	CHIS					Non-CHIS				
	wage			PW		wage		PW		
BC	−0.2255 *** (0.0367)			−0.0625 * (0.0319)		0.0473 (0.0323)		−0.0559 ** (0.0277)		
BC × wage				0.0020 (0.0012)				0.0016 *** (0.0011)		
**Panel C. OECD vs. Non-OECD**										
	OECD					Non-OECD				
	wage			PW		wage		PW		
BC	−0.0076 (0.0170)			−0.0864 *** (0.0293)		−0.1583 *** (0.0486)		−0.0667 ** (0.0287)		
BC × wage				0.0030 ** (0.00127)				0.0019 ** (0.0011)		
**Panel D. World Bank High Income vs. Middle/Low Income Level**										
	High income					Middle/Low income				
	wage			PW		wage		PW		
BC	−0.0318 (0.0214)			−0.0706 *** (0.0271)		−0.1331 ** (0.0514)		−0.1205 *** (0.0337)		
BC × wage				0.0025** (0.0011)				0.0038 *** (0.0013)		
**Panel E. Regional Clustering**										
	Africa		Asia		Europe		Latin America		Anglo-Saxon (excluding UK)	
	wage	PW	wage	PW	wage	PW	wage	PW	wage	PW
BC	−0.1730 * (0.0900)	−0.1958 * (0.1026)	−0.1862 *** (0.0461)	0.0480 (0.0319)	−0.0236 (0.0131)	−0.1470 ** (0.0598)	0.0268 (0.1064)	−0.1059 ** (0.0481)	−0.0356 (0.0232)	−0.1884 ** (0.0892)
BC × wage		0.0075 * (0.0041)		0.0013 (0.0012)		0.0054 ** (0.0025)		0.0032 * (0.0018)		0.0073 ** (0.0037)
Control variables	YES									
Virtual variable	YES									

Note: Panel A is the estimated result of the full sample. Panel B divides the full sample into two sub-samples, that is, CHIS and Non-CHIS countries. Panel C divides the full sample into two sub-samples, that is, OECD and Non-OECD countries. Panel D divides the full sample into high-income and low-middle-income countries according to the World Bank’s classification criteria. Panel E is divided by regions. BC and FC represent economic recession in the economic cycle and financial crisis respectively. All models incorporate control variables and treat countries as dummy variables. Each subsample gives two columns of empirical results. The first column of explanatory variables includes economic recession variables and control variables, and the latter column introduces interaction variables of economic recession and financial crisis. Standard errors in parentheses, *** *p* < 0.01, ** *p* < 0.05, * *p* < 0.1.

## Appendix A

**Table A1:** Country classification.

Country	Health Systems Classification	OECD Classification	World Bank Income Lever Classification (2018)	World’s Region Classification
ARG	Non-CHIS	Non-OECD	Middle/Low income level	Latin-America
AUS	Non-CHIS	OECD	High income level	Anglo-Saxon
AUT	Non-CHIS	OECD	High income level	Europe
BEL	Non-CHIS	OECD	High income level	Europe
BGD	Non-CHIS	Non-OECD	Middle/Low income level	Asia
BRA	CHIS	Non-OECD	Middle/Low income level	Latin-America
CAF	Non-CHIS	Non-OECD	Middle/Low income level	Africa
CAN	CHIS	OECD	High income level	Anglo-Saxon
CHE	Non-CHIS	OECD	High income level	Europe
CHL	CHIS	OECD	High income level	Latin-America
CHN	Non-CHIS	Non-OECD	Middle/Low income level	Asia
COL	CHIS	Non-OECD	Middle/Low income level	Latin-America
CRI	CHIS	Non-OECD	Middle/Low income level	Latin-America
DEU	Non-CHIS	OECD	High income level	Europe
DNK	CHIS	OECD	High income level	Europe
DZA	Non-CHIS	Non-OECD	Middle/Low income level	Asia
ECU	CHIS	Non-OECD	Middle/Low income level	Latin-America
EGY	CHIS	Non-OECD	Middle/Low income level	Africa
ESP	CHIS	OECD	High income level	Europe
FIN	CHIS	OECD	High income level	Europe
FRA	Non-CHIS	OECD	High income level	Europe
GBR	CHIS	OECD	High income level	Europe
GRC	CHIS	OECD	High income level	Europe
IDN	Non-CHIS	Non-OECD	Middle/Low income level	Asia
IND	CHIS	Non-OECD	Middle/Low income level	Asia
IRL	CHIS	OECD	High income level	Europe
IRN	Non-CHIS	Non-OECD	Middle/Low income level	Asia
ISR	Non-CHIS	OECD	High income level	Asia
ITA	CHIS	OECD	High income level	Europe
JPN	CHIS	OECD	High income level	Asia
KAZ	CHIS	Non-OECD	Middle/Low income level	Asia
KEN	CHIS	Non-OECD	Middle/Low income level	Africa
KOR	CHIS	OECD	High income level	Asia
KWT	CHIS	Non-OECD	High income level	Asia
LKA	Non-CHIS	Non-OECD	Middle/Low income level	Asia
MAR	CHIS	Non-OECD	Middle/Low income level	Africa
MEX	Non-CHIS	OECD	Middle/Low income level	Latin-America
MYS	CHIS	Non-OECD	Middle/Low income level	Asia
NGA	Non-CHIS	Non-OECD	Middle/Low income level	Africa
NLD	Non-CHIS	OECD	High income level	Europe
NOR	CHIS	OECD	High income level	Europe
NZL	Non-CHIS	OECD	High income level	Anglo-Saxon
PAK	CHIS	Non-OECD	Middle/Low income level	Asia
PER	Non-CHIS	Non-OECD	Middle/Low income level	Latin-America
PHL	Non-CHIS	Non-OECD	Middle/Low income level	Asia
POL	Non-CHIS	OECD	High income level	Europe
PRT	CHIS	OECD	High income level	Europe
PRY	Non-CHIS	Non-OECD	Middle/Low income level	Latin-America
RUS	CHIS	Non-OECD	Middle/Low income level	Europe
SAU	Non-CHIS	Non-OECD	High income level	Asia
SDN	Non-CHIS	Non-OECD	Middle/Low income level	Africa
SEN	Non-CHIS	Non-OECD	Middle/Low income level	Africa
SGP	Non-CHIS	Non-OECD	High income level	Asia
SWE	Non-CHIS	OECD	High income level	Europe
THA	CHIS	Non-OECD	Middle/Low income level	Asia
TUR	Non-CHIS	OECD	Middle/Low income level	Asia
URY	Non-CHIS	Non-OECD	High income level	Latin-America
USA	Non-CHIS	OECD	High income level	Anglo-Saxon
VEN	Non-CHIS	Non-OECD	Middle/Low income level	Latin-America
ZAF	Non-CHIS	Non-OECD	Middle/Low income level	Africa

Note: Countries classified according to three different labels: (1) CHIS vs. non-CHIS memberships; (2) OECD vs. non-OECD memberships; (3) World Bank Income Level Classification from 2018; and (4) Region or political/cultural association.
